# *N*-(3-Nitro­benzyl­idene)aniline

**DOI:** 10.1107/S1600536808036970

**Published:** 2008-11-20

**Authors:** Muhammad Zaheer, Zareen Akhter, Michael Bolte, Humaira M. Siddiqi

**Affiliations:** a Department of Chemistry, Quaid-I-Azam University, Islamabad 45320, Pakistan; b Institut für Anorganische Chemie, J. W. Goethe-Universität Frankfurt, Max-von-Laue-Strasse 7, 60438 Frankfurt/Main, Germany

## Abstract

In the title compound, C_13_H_10_N_2_O_2_, a Schiff base derivative, the dihedral angle between the two aromatic rings is 31.58 (3)°. The C=N double bond is essentially coplanar with the nitro­phenyl ring. The torsion angle of the imine double bond is 175.97 (13)°, indicating that the C=N double bond is in a *trans* configuration. The crystal structure is stabilized by C—H⋯O contacts and π–π inter­actions (centroid–centroid distances of 3.807 and 3.808Å).

## Related literature

Choi *et al.* (2000 ▶) and Nakamura *et al.* (1999 ▶) discuss the use of Schiff bases in the reduction of thionyl chloride, while Maruyama *et al.* (1995 ▶) and Burrows *et al.* (1996 ▶) describe their use in degradation processes. Hodnett & Mooney (1970 ▶), Rajavel *et al.* (2008 ▶) and Yu *et al.* (2007 ▶) discuss anti­neoplastic, anti­bacterial and anti­fungal activities, respectively. Hartley *et al.* (2002 ▶), Torregrosa *et al.* (2005 ▶) and Naeimi *et al.* (2008 ▶) describe different synthetic routes towards Schiff bases. Landy (1989 ▶) describes their role in biological redox systems. Yoon *et al.* (1990 ▶) and Park *et al.* (1998 ▶) discuss properties of Schiff base complexes such as alkene epoxidation and oxygen absorption by cobalt(II) complexes. Flack (1983 ▶) discusses the Rogers’s parameter for the characterization of enanti­o­morphic-polar compounds.
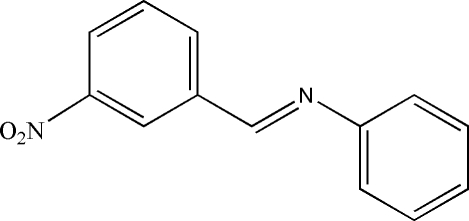


## Experimental

### 

#### Crystal data

C_13_H_10_N_2_O_2_
*M* *_r_* = 226.23
Orthorhombic, 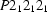

*a* = 7.3177 (6) Å
*b* = 12.1022 (11) Å
*c* = 12.4672 (12) Å
*V* = 1104.10 (17) Å^3^
*Z* = 4
Mo *K*α radiation
μ = 0.09 mm^−1^
*T* = 173 (2) K
0.48 × 0.48 × 0.46 mm

#### Data collection

Stoe IPDSII two-circle diffractometer
Absorption correction: none
9868 measured reflections
1585 independent reflections
1421 reflections with *I* > 2σ(*I*)
*R*_int_ = 0.053

#### Refinement

*R*[*F* ^2^ > 2σ(*F* ^2^)] = 0.034
*wR*(*F* ^2^) = 0.093
*S* = 1.04
1585 reflections
154 parameters
H-atom parameters constrained
Δρ_max_ = 0.26 e Å^−3^
Δρ_min_ = −0.14 e Å^−3^

### 

Data collection: *X-AREA* (Stoe & Cie, 2001 ▶); cell refinement: *X-AREA*; data reduction: *X-AREA*; program(s) used to solve structure: *SHELXS97* (Sheldrick, 2008 ▶); program(s) used to refine structure: *SHELXL97* (Sheldrick, 2008 ▶); molecular graphics: *XP* in *SHELXTL-Plus* (Sheldrick, 2008 ▶); software used to prepare material for publication: *SHELXL97*.

## Supplementary Material

Crystal structure: contains datablocks I, global. DOI: 10.1107/S1600536808036970/zl2143sup1.cif

Structure factors: contains datablocks I. DOI: 10.1107/S1600536808036970/zl2143Isup2.hkl

Additional supplementary materials:  crystallographic information; 3D view; checkCIF report

## Figures and Tables

**Table 1:** Hydrogen-bond geometry (Å, °)

*D*—H⋯*A*	*D*—H	H⋯*A*	*D*⋯*A*	*D*—H⋯*A*
C6—H6⋯O1^i^	0.95	2.70	3.261 (2)	118
C6—H6⋯O2^ii^	0.95	2.71	3.303 (2)	122
C7—H7⋯O1^i^	0.95	2.66	3.237 (2)	120
C13—H13⋯O2^iii^	0.95	2.64	3.541 (2)	159

Symmetry codes: (i) 
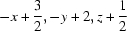
; (ii) 
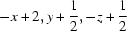
; (iii) 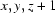.

**Fig. 1.**
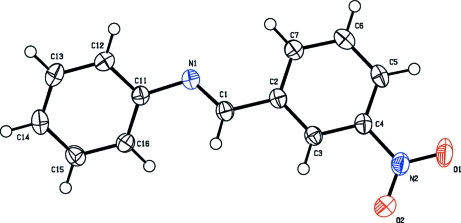
Perspective view of the title compound with the numbering scheme and displacement ellipsoids at the 50 % probability level. H atoms are drawn as spheres of arbitrary radii.

**Fig. 2.**
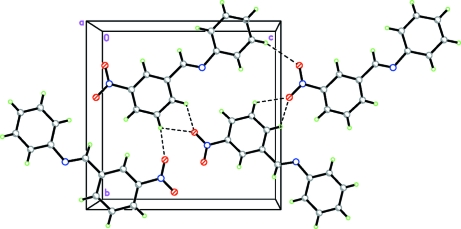
Packing diagram of the title compound with view onto the bc plane. Hydrogen bonds are shown as dashed lines.

**Fig. 3.**
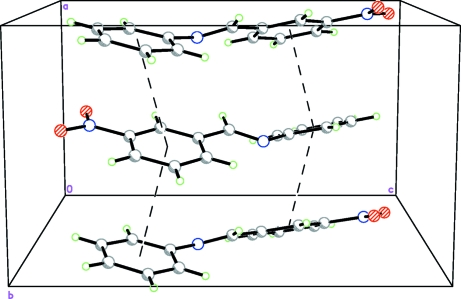
Packing diagram of the title compound with view onto the ac plane. π–π stacking interactions are shown as dashed lines.

## supplementary crystallographic information

### Comment

Schiff bases and their complexes are widely studied because of their interesting and important properties such as their ability to reversibly bind oxygen (Park *et al.*, 1998) and their use in catalysis. They are part of redox systems in biological systems (Landy, 1989), they are used in the degradation of dyes through decomposition of hydrogen peroxide and other reagents in the textile industry (Maruyama *et al.*, 1995) as well as in the reduction of thionyl chloride (Choi *et al.*, 2000;  Nakamura *et al.*, 1999). These compounds can also be used in the degradation of organic compounds (Burrows *et al.*, 1996) and in radiopharmaceuticals (Yoon *et al.*, 1990). Schiff bases also exhibit antineoplastic (Hodnett *et al.*, 1970) antibacterial (Rajavel *et al.*, 2008) and antifungal (Yu *et al.* 2007) activities. The compound whose crystal structure is reported was synthesized for comparative studies of the biological applications of Schiff bases with and without a ferrocene moiety. The synthesis of the present compound was reported earlier by Torregrosa (Torregrosa *et al.*, 2005), Hartley (Hartley *et al.*, 2002) and Naeimi (Naeimi *et al.*, 2008). We have utilized a different modified method for the synthesis of this compound as described below.

Geometric parameters of the title compound (Fig. 1) are in the usual ranges. The molecule is composed of two almost planar moieties, the 3-nitrobenzylidene moieties and the phenyl ring. The dihedral angle between the two aromatic rings is 31.58 (3)°. The torsion angle C11-N1-C1-C2 [175.97 (13)°] shows that the C-N double bond is trans configured. The crystal packing (Fig. 2) is stabilized by some short C-H···O contacts (see Table 1) and π–π stacking interactions (cog_phenyl_···cog_nitrophenyl_^i^ = 3.807Å, cog_phenyl_···cog_nitrophenyl_^ii^ = 3.808Å; symmetry operators: (i) -1/2+x, 3/2-y, 1-z; (ii) 1/2+x, 3/2-y, 1-z).

### Experimental

In a 250 ml pre-backed two neck flask supplied with a magnetic stirrer, 4 ml (43 mmol) of freshly distilled aniline was mixed with 4.97 g (43 mmol) of 3-nitrobenzaldehyde in dry toluene as the solvent. The reaction mnixture was heated to reflux using a Dean and Stark apparatus for azeotropic removal of water formed during the reaction. Reaction progress was monitored using TLC and the solid obtained after rotary evaporation was recrystallized from a mixture of ethyl acetate and n-hexane. Yield: 80%, melting point: 337-338K.

### Refinement

All H atoms could be located by difference Fourier synthesis. Nevertheless, they were refined with fixed individual isotropic displacement parameters [U_iso_(H) = 1.2 *U*_eq_(C)] using a riding model with C—H = 0.95 Å.

In the absence of anomalous scatterers, the Flack (1983) parameter is meaningless and therefore Friedel pairs were merged prior to refinement.

### Crystal data

**Table:**

C_13_H_10_N_2_O_2_	*F*_000_ = 472
*M**_r_* = 226.23	*D*_x_ = 1.361 Mg m^−^^3^
Orthorhombic, *P*2_1_2_1_2_1_	Mo *K*α radiation λ = 0.71073 Å
Hall symbol: P 2ac 2ab	Cell parameters from 7899 reflections
*a* = 7.3177 (6) Å	θ = 3.7–25.8º
*b* = 12.1022 (11) Å	µ = 0.09 mm^−^^1^
*c* = 12.4672 (12) Å	*T* = 173 (2) K
*V* = 1104.10 (17) Å^3^	Block, colourless
*Z* = 4	0.48 × 0.48 × 0.46 mm

### Data collection

**Table:**

Stoe IPDSII two-circle diffractometer	1421 reflections with *I* > 2σ(*I*)
Radiation source: fine-focus sealed tube	*R*_int_ = 0.054
Monochromator: graphite	θ_max_ = 29.5º
*T* = 173(2) K	θ_min_ = 3.6º
ω scans	*h* = −9→8
Absorption correction: none	*k* = −16→14
9868 measured reflections	*l* = −15→17
1585 independent reflections	

### Refinement

**Table:**

Refinement on *F*^2^	Secondary atom site location: difference Fourier map
Least-squares matrix: full	Hydrogen site location: inferred from neighbouring sites
*R*[*F*^2^ > 2σ(*F*^2^)] = 0.034	H-atom parameters constrained
*wR*(*F*^2^) = 0.093	*w* = 1/[σ^2^(*F*_o_^2^) + (0.0638*P*)^2^ + 0.081*P*] where *P* = (*F*_o_^2^ + 2*F*_c_^2^)/3
*S* = 1.04	(Δ/σ)_max_ < 0.001
1585 reflections	Δρ_max_ = 0.26 e Å^−^^3^
154 parameters	Δρ_min_ = −0.14 e Å^−^^3^
Primary atom site location: structure-invariant direct methods	Extinction correction: none

### Special details

**Table:**

Geometry. All e.s.d.'s (except the e.s.d. in the dihedral angle between two l.s. planes) are estimated using the full covariance matrix. The cell e.s.d.'s are taken into account individually in the estimation of e.s.d.'s in distances, angles and torsion angles; correlations between e.s.d.'s in cell parameters are only used when they are defined by crystal symmetry. An approximate (isotropic) treatment of cell e.s.d.'s is used for estimating e.s.d.'s involving l.s. planes.
Refinement. Refinement of *F*^2^ against ALL reflections. The weighted *R*-factor *wR* and goodness of fit *S* are based on *F*^2^, conventional *R*-factors *R* are based on *F*, with *F* set to zero for negative *F*^2^. The threshold expression of *F*^2^ > σ(*F*^2^) is used only for calculating *R*-factors(gt) *etc*. and is not relevant to the choice of reflections for refinement. *R*-factors based on *F*^2^ are statistically about twice as large as those based on *F*, and *R*- factors based on ALL data will be even larger.

### Fractional atomic coordinates and isotropic or equivalent isotropic displacement parameters (Å^2^)

**Table:**

	*x*	*y*	*z*	*U*_iso_*/*U*_eq_	
N1	0.85150 (17)	0.74236 (10)	0.59232 (10)	0.0265 (3)	
N2	0.9518 (2)	0.83182 (11)	0.11436 (10)	0.0339 (3)	
O1	0.9459 (2)	0.90457 (11)	0.04548 (10)	0.0507 (4)	
O2	0.9936 (2)	0.73588 (10)	0.09536 (9)	0.0525 (4)	
C1	0.9027 (2)	0.72475 (11)	0.49536 (12)	0.0255 (3)	
H1	0.9530	0.6549	0.4771	0.031*	
C2	0.8855 (2)	0.81012 (11)	0.41137 (12)	0.0239 (3)	
C3	0.9276 (2)	0.78262 (11)	0.30530 (11)	0.0243 (3)	
H3	0.9685	0.7104	0.2876	0.029*	
C4	0.9088 (2)	0.86252 (12)	0.22632 (11)	0.0262 (3)	
C5	0.8499 (2)	0.96943 (12)	0.24807 (12)	0.0296 (3)	
H5	0.8383	1.0225	0.1924	0.036*	
C6	0.8086 (2)	0.99625 (12)	0.35358 (13)	0.0314 (4)	
H6	0.7678	1.0687	0.3705	0.038*	
C7	0.8262 (2)	0.91791 (12)	0.43499 (12)	0.0277 (3)	
H7	0.7979	0.9375	0.5069	0.033*	
C11	0.86161 (19)	0.65339 (11)	0.66706 (11)	0.0239 (3)	
C12	0.9017 (2)	0.67860 (12)	0.77429 (12)	0.0291 (3)	
H12	0.9229	0.7531	0.7947	0.035*	
C13	0.9105 (2)	0.59535 (14)	0.85102 (12)	0.0337 (4)	
H13	0.9399	0.6130	0.9232	0.040*	
C14	0.8763 (2)	0.48619 (13)	0.82226 (13)	0.0332 (4)	
H14	0.8832	0.4292	0.8745	0.040*	
C15	0.8317 (2)	0.46086 (12)	0.71627 (13)	0.0308 (3)	
H15	0.8062	0.3866	0.6968	0.037*	
C16	0.8244 (2)	0.54339 (12)	0.63890 (12)	0.0261 (3)	
H16	0.7942	0.5253	0.5669	0.031*	

### Atomic displacement parameters (Å^2^)

**Table:**

	*U*^11^	*U*^22^	*U*^33^	*U*^12^	*U*^13^	*U*^23^
N1	0.0264 (6)	0.0264 (6)	0.0267 (6)	−0.0005 (5)	0.0001 (5)	0.0030 (5)
N2	0.0391 (8)	0.0365 (7)	0.0262 (6)	−0.0015 (6)	−0.0042 (6)	0.0052 (5)
O1	0.0677 (9)	0.0548 (7)	0.0297 (6)	0.0073 (8)	0.0022 (7)	0.0184 (5)
O2	0.0883 (12)	0.0392 (7)	0.0301 (6)	0.0053 (7)	0.0001 (7)	−0.0027 (5)
C1	0.0230 (7)	0.0265 (7)	0.0271 (7)	0.0011 (6)	−0.0012 (6)	0.0032 (6)
C2	0.0210 (6)	0.0249 (6)	0.0259 (7)	−0.0010 (5)	−0.0021 (6)	0.0033 (5)
C3	0.0227 (7)	0.0242 (6)	0.0261 (6)	−0.0004 (5)	−0.0030 (6)	0.0026 (5)
C4	0.0255 (7)	0.0278 (7)	0.0255 (7)	−0.0031 (6)	−0.0027 (6)	0.0043 (6)
C5	0.0279 (8)	0.0267 (7)	0.0342 (7)	−0.0009 (6)	−0.0048 (6)	0.0093 (6)
C6	0.0304 (8)	0.0239 (7)	0.0399 (8)	0.0012 (6)	−0.0006 (7)	0.0031 (6)
C7	0.0257 (7)	0.0270 (7)	0.0305 (7)	0.0009 (6)	0.0022 (6)	0.0023 (6)
C11	0.0204 (6)	0.0264 (6)	0.0248 (7)	0.0013 (5)	0.0026 (6)	0.0024 (6)
C12	0.0297 (8)	0.0313 (7)	0.0264 (7)	0.0001 (6)	0.0013 (6)	−0.0029 (6)
C13	0.0351 (8)	0.0439 (8)	0.0222 (6)	0.0014 (7)	−0.0008 (7)	0.0021 (6)
C14	0.0306 (8)	0.0376 (8)	0.0314 (7)	0.0023 (6)	0.0023 (6)	0.0127 (7)
C15	0.0297 (8)	0.0269 (7)	0.0359 (8)	−0.0003 (6)	0.0037 (7)	0.0032 (6)
C16	0.0252 (7)	0.0283 (7)	0.0247 (6)	−0.0001 (6)	0.0015 (6)	−0.0001 (6)

### Geometric parameters (Å, °)

**Table:**

N1—C1	1.283 (2)	C6—C7	1.395 (2)
N1—C11	1.4258 (18)	C6—H6	0.9500
N2—O2	1.2237 (18)	C7—H7	0.9500
N2—O1	1.2306 (17)	C11—C12	1.402 (2)
N2—C4	1.4783 (19)	C11—C16	1.4034 (19)
C1—C2	1.476 (2)	C12—C13	1.391 (2)
C1—H1	0.9500	C12—H12	0.9500
C2—C3	1.398 (2)	C13—C14	1.391 (2)
C2—C7	1.4060 (19)	C13—H13	0.9500
C3—C4	1.3869 (19)	C14—C15	1.395 (2)
C3—H3	0.9500	C14—H14	0.9500
C4—C5	1.390 (2)	C15—C16	1.390 (2)
C5—C6	1.388 (2)	C15—H15	0.9500
C5—H5	0.9500	C16—H16	0.9500
			
C1—N1—C11	118.36 (12)	C6—C7—C2	120.45 (14)
O2—N2—O1	123.54 (14)	C6—C7—H7	119.8
O2—N2—C4	118.32 (12)	C2—C7—H7	119.8
O1—N2—C4	118.14 (13)	C12—C11—C16	119.04 (13)
N1—C1—C2	121.80 (13)	C12—C11—N1	118.00 (12)
N1—C1—H1	119.1	C16—C11—N1	122.88 (13)
C2—C1—H1	119.1	C13—C12—C11	120.52 (14)
C3—C2—C7	119.15 (12)	C13—C12—H12	119.7
C3—C2—C1	119.04 (12)	C11—C12—H12	119.7
C7—C2—C1	121.81 (13)	C12—C13—C14	120.15 (14)
C4—C3—C2	118.92 (13)	C12—C13—H13	119.9
C4—C3—H3	120.5	C14—C13—H13	119.9
C2—C3—H3	120.5	C13—C14—C15	119.65 (14)
C3—C4—C5	122.76 (14)	C13—C14—H14	120.2
C3—C4—N2	118.30 (13)	C15—C14—H14	120.2
C5—C4—N2	118.94 (12)	C16—C15—C14	120.57 (14)
C6—C5—C4	118.03 (13)	C16—C15—H15	119.7
C6—C5—H5	121.0	C14—C15—H15	119.7
C4—C5—H5	121.0	C15—C16—C11	120.04 (13)
C5—C6—C7	120.69 (14)	C15—C16—H16	120.0
C5—C6—H6	119.7	C11—C16—H16	120.0
C7—C6—H6	119.7		
			
C11—N1—C1—C2	175.97 (13)	C5—C6—C7—C2	−0.3 (2)
N1—C1—C2—C3	−174.08 (15)	C3—C2—C7—C6	0.4 (2)
N1—C1—C2—C7	5.3 (2)	C1—C2—C7—C6	−179.00 (14)
C7—C2—C3—C4	−0.3 (2)	C1—N1—C11—C12	146.81 (14)
C1—C2—C3—C4	179.08 (12)	C1—N1—C11—C16	−36.5 (2)
C2—C3—C4—C5	0.1 (2)	C16—C11—C12—C13	2.1 (2)
C2—C3—C4—N2	−179.28 (15)	N1—C11—C12—C13	179.00 (13)
O2—N2—C4—C3	3.5 (2)	C11—C12—C13—C14	−1.1 (2)
O1—N2—C4—C3	−175.59 (15)	C12—C13—C14—C15	−0.5 (2)
O2—N2—C4—C5	−175.94 (16)	C13—C14—C15—C16	1.1 (2)
O1—N2—C4—C5	5.0 (2)	C14—C15—C16—C11	−0.1 (2)
C3—C4—C5—C6	0.0 (2)	C12—C11—C16—C15	−1.5 (2)
N2—C4—C5—C6	179.38 (14)	N1—C11—C16—C15	−178.20 (13)
C4—C5—C6—C7	0.1 (2)		

### Hydrogen-bond geometry (Å, °)

**Table:**

*D*—H···*A*	*D*—H	H···*A*	*D*···*A*	*D*—H···*A*
C6—H6···O1^i^	0.95	2.70	3.261 (2)	118
C6—H6···O2^ii^	0.95	2.71	3.303 (2)	122
C7—H7···O1^i^	0.95	2.66	3.237 (2)	120
C13—H13···O2^iii^	0.95	2.64	3.541 (2)	159

Symmetry codes: (i) −*x*+3/2, −*y*+2, *z*+1/2; (ii) −*x*+2, *y*+1/2, −*z*+1/2; (iii) *x*, *y*, *z*+1.
